# 基于氧化石墨烯气凝胶固相萃取柱检测有机磷农药

**DOI:** 10.3724/SP.J.1123.2021.03032

**Published:** 2022-01-08

**Authors:** Xiudan HOU, Hui YU, Feng ZHU, Zhaojie LI, Qingli YANG

**Affiliations:** 1.青岛农业大学食品科学与工程学院, 山东 青岛 266109; 1. College of Food Science and Engineering, Qingdao Agricultural University, Qingdao 266109, China; 2.青岛市即墨区综合检验检测中心, 山东 青岛 266200; 2. Jimo Comprehensive Inspection and Testing Center, Qingdao 266200, China

**Keywords:** 高效液相色谱, 固相萃取, 氧化石墨烯气凝胶, 有机磷农药, high performance liquid chromatography (HPLC), solid phase extraction (SPE), graphene oxide aerogel, organophosphorus pesticides

## Abstract

以氧化石墨烯气凝胶三维纳米材料作为固相萃取的吸附剂,结合高效液相色谱,对食品中的有机磷农药(辛硫磷、双硫磷、倍硫磷、杀螟硫磷)进行检测分析。首先,利用冷冻干燥的方式制备得到氧化石墨烯气凝胶,通过扫描电镜、红外光谱、比表面积吸附等一系列的实验手段对其形貌及物理特性进行了表征,证明其成功合成。从扫描电镜中可见石墨烯的层状褶皱结构,其表面积为740.51 m^2^/g。然后,将氧化石墨烯气凝胶直接填充于固相萃取柱中,在未借助任何硅胶等基体的条件下进行萃取研究;通过单因素实验,系统研究了萃取和洗脱条件对有机磷农药萃取回收率的影响。结果显示,在上样体积15 mL、样品溶液pH值4、上样速率1.0 mL/min、洗脱剂1.0 mL乙腈的条件下萃取回收率最高。与商用的萃取材料进行比较,包括碳十八硅胶柱(C18)、阴离子交换柱(SAX)、氨基柱(-NH_2_)和硅酸镁柱(Florisil),氧化石墨烯气凝胶填充的固相萃取柱的萃取回收率有明显提高。实验考察了氧化石墨烯气凝胶直接填充的萃取柱的寿命,结果显示该萃取柱可以重复使用15次,可见解决了分散无基体支撑的石墨烯纳米片容易破碎、堵塞筛板的问题。与液相色谱联用建立分析方法,4种有机磷农药的线性范围较宽,辛硫磷、双硫磷和倍硫磷的线性范围为1~200 μg/L,杀螟硫磷的线性范围为2~200 μg/L,线性拟合良好(线性相关系数*r*^2^≥0.9949),检出限为0.2~0.5 μg/L,满足于我国和其他国家限定标准的检测。将该方法应用于实际样品,在苹果皮中未检测到有机磷农药,对其进行加标,回收率为70.5%~93.6%,相对标准偏差≤10.4%。

## Abstract (en)

A graphene oxide aerogel was prepared and directly filled in a solid phase extraction (SPE) column without the aid of silica or other substrates. The aerogel was used to extract and detect residual organophosphorus pesticides (phoxim, temephos, fenthion, and fenitrothion) in food, and exhibited good elasticity and high mechanical strength. The graphene oxide aerogel was prepared by freeze-drying. Its morphology and physical properties were characterized by scanning electron microscopy, infrared spectroscopy, and BET surface adsorption. Results proved the successful synthesis of the graphene oxide aerogel. Scanning electron micrographs of the aerogel exhibited a layered and fold structure, with a surface area of 740.51 m^2^/g. The effect of experimental conditions on the extraction recovery of organophosphorus pesticides was systematically studied through a series of single-factor experiments. Due to limited adsorption sites, sample volumes of 5-60 mL were investigated, and 15 mL was determined was the optimum sample volume. The rate of sample loading was investigated in the range of 0.8-3.0 mL/min. When the rate of sample loading was higher than 3.0 mL/min, the insufficient contact between the analytes and sorbent appeared to cause a decrease in the extraction recovery. A lower rate of sample loading would prolong the operation time due to the re-elution of organophosphorus pesticides. The sample pH was optimized from a pH range of 2-11. An acidic solution was found to be good for inducing electrostatic interactions between the graphene oxide aerogel and organophosphorus pesticides. The maximum extraction recoveries were obtained at pH 4. Three eluents (acetonitrile, methanol, and acetone) were explored for optimization, and results showed that acetonitrile was the most appropriate eluent. The eluent volume (0.6-1.2 mL) was also investigated, and the optimal value was found to be 1.0 mL. Compared with commercial extraction materials including C18 silica, the anion exchange column (SAX), amino (-NH_2_), and Florisil, the extraction recovery of this new material showed distinct improvement. The lifetime of the extraction column directly filled with the graphene oxide aerogel was investigated. The column could be repeatedly used for 15 times, which overcame the issue of blocking of the sieve plates of fragmented graphene nanosheets dispersed without any matrix support. The linearities of the four organophosphorus pesticides were 1-200 μg/L for phoxim, temephos, and fenthion, and 2-200 μg/L for fenitrothion. The linear correlation coefficients were all ≥0.9949, and limits of detection were in the range of 0.2-0.5 μg/L. An extraction column was used to extract the analytes continuously for five times, and the RSDs of the extraction recoveries were ≤6.5%. Subsequently, five extraction columns were used to extract the analytes under the same conditions, and the RSDs of the extraction recoveries were ≤11.3%. Finally, the established method was applied for the extraction and detection of a real sample (apple peel); no organophosphorus pesticide was detected in the apple peel. The recoveries for spiked standard solutions were between 70.5% and 93.6%, and RSDs were ≤10.4%.

有机磷农药是一类含磷的有机化合物,具有高效的杀虫效果,在农业生产中得到广泛应用,但过量的残留会引起水、空气、土壤以及蔬菜、水果和加工食品的污染,对生态环境和人类健康造成极大的伤害^[1]^。据统计,我国因食用农药污染的食品而引起中毒的人数约占食物中毒总人数的1/3,其中有机磷农药引起的食物中毒现象占据我国农药食物中毒的第一位。目前有机磷农药的国家检测标准存在耗时长、效率低、检出限高等问题^[2]^。因此,开发针对有机磷农药准确、快速、灵敏度高、检出限低的分析方法对保障食品安全具有重要意义^[3]^。

有机磷农药残留的检测方法主要有气相色谱-质谱法和高效液相色谱-质谱法等^[4,5]^。由于食品基质的复杂性、成分的多样性以及农药残留的痕量性,仪器很难直接检测到食品中痕量的有机磷农药。为了提高灵敏度和准确性,有必要采用合适的样品前处理方法对其分离富集,然后进样检测^[6,7]^。近年来,有机磷农药检测中使用的基于纳米材料的萃取方法,主要包括分散液液萃取、中空纤维液相萃取、分散固相萃取、磁固相萃取等^[8,9,10,11,12]^;使用的纳米材料主要包括金属有机框架材料、共价有机框架材料、离子液体、金属硫化物等^[13,14,15,16,17]^。

固相萃取作为一种高效的样品前处理技术,它实现对痕量分析物富集浓缩的同时,还可以去除样品基质,消除干扰^[18,19,20,21]^。石墨烯自问世以来,在样品前处理领域得到极大关注^[22]^,之前有研究^[23]^将石墨烯纳米片直接填充于带有筛板的固相萃取小柱中,但是分散无基体支撑的石墨烯纳米片容易破碎,堵塞筛板。也有一些研究^[24]^将石墨烯固定到硅胶载体上,但方法相对繁琐,组装量较少。最近,有机和无机气凝胶材料受到很大关注^[25,26]^,石墨烯气凝胶作为一种三维石墨烯材料,自问世以来,由于超大的比表面积、良好的机械性能、极大的孔隙率、极低的密度等优点,被广泛应用于不同研究领域中^[27,28,29,30,31,32]^。石墨烯气凝胶具有弹性,机械强度强,可作为吸附剂被直接填充于固相萃取小柱中,不易破碎;除此之外,石墨烯气凝胶具有三维框架结构,一定程度上可以避免石墨烯片层之间的堆积。

本实验以氧化石墨烯气凝胶为吸附剂构建固相萃取体系,建立了一种对有机磷农药高效的样品前处理方法,结合HPLC-DAD,对苹果皮中4种有机磷农药进行萃取检测,并通过考察加标回收率,分析食品中基质的影响。

## 1 实验部分

### 1.1 仪器、试剂与材料

UltiMate3000高效液相色谱仪配有高压四元泵、自动脱气装置、自动进样器、柱温箱、DAD检测器(美国Thermo公司); Nicolet Nexus 870傅里叶变换红外光谱仪(美国Nicolet公司); JSM-6701F扫描电子显微镜(日本电子株式会社); SPE-80数控固相萃取仪(济南海能仪器股份有限公司); Millipore Direct-Q纯水仪(18.2 MΩ·cm,美国默克密理博公司); New Classic MF分析天平(天津市德安特传感技术有限公司); KQ-500TDE型高频数控超声波清洗器(昆山市超声仪器有限公司); TGL-16GB高速离心机(上海安亭科学仪器厂); FW-80高速万能粉碎机(天津市泰斯特仪器有限公司)。

辛硫磷、双硫磷、倍硫磷、杀螟硫磷(纯度>99%,上海阿拉丁试剂公司);石墨粉、五氧化二磷购自上海麦克林生化科技有限公司;甲醇、乙醇(天津市科密欧化学试剂有限公司);过硫酸钾(天津市巴斯夫化工有限公司);浓硫酸、高锰酸钾、盐酸(莱阳市康德化工有限公司); 30%过氧化氢(西陇化工试剂有限公司)。

### 1.2 实验条件

1.2.1 材料的制备

采用Hummers法^[9]^对天然石墨粉进行一系列处理制备得到氧化石墨烯。将10 g五氧化二磷和10 g过硫酸钾混合加入锥形瓶中,加热至80 ℃,再加入60 mL硫酸,持续一段时间后,加入40 g石墨粉,继续加热2 h,冷却至室温,抽滤。将20 mL硫酸加入到容积为2 L的大烧杯中,保持10 ℃以下加入2 g抽滤好的石墨,再加入1 g硝酸钠,1 h后进行超声处理。30 min后,加入3 g高锰酸钾,保持低温2 h。超声处理30 min后,加入200 mL蒸馏水,保持95 ℃ 20 min。加入150 mL超纯水、30 mL过氧化氢、80 mL盐酸,保持20 min,然后离心(5000 r/min)10 min,倒入2 L超纯水中,得到氧化石墨烯悬浮液。

将上述得到的氧化石墨烯悬浮液放入-80 ℃超低温冰箱中冷冻2 h,随后转移至冷冻干燥机中,在20 Pa、-50 ℃条件下经8 h后得到氧化石墨烯气凝胶。

1.2.2 标准溶液配制

有机磷农药标准品的储备液均由甲醇配制,质量浓度为0.2 mg/mL,置于4 ℃冰箱中保存。使用之前,采用一级蒸馏水将储备液稀释至0.1、0.2、0.5、0.8、1、2、5、10、20、50、100、200 μg/L,配制成系列浓度的标准溶液。

1.2.3 样品前处理

从当地市场(中国青岛)购买了烟台红富士苹果。首先,使用高速粉碎机将苹果皮粉碎成粉末,将1 g苹果皮粉末置于10 mL乙醇-水(70∶30, v/v)中,于55 ℃超声萃取3 h。之后,通过离心将苹果皮和萃取剂分离,将萃取剂在氮气流下蒸发。最后,将残留物用20 mL超纯水重溶,以进行萃取。

1.2.4 固相萃取过程

将5 mg氧化石墨烯气凝胶填充于3 mL固相萃取空柱中,利用筛板将其固定。首先,使用5 mL乙腈和5 mL水依次通过固相萃取小柱淋洗活化材料,移取15 mL样品溶液以1.0 mL/min流速进行上样;完成上样过程后,用0.5 mL水将吸附在萃取剂表面的杂质冲洗下来,然后将萃取小柱置于空气中干燥5 min,随即利用1.0 mL乙腈以0.6 mL/min的速率对吸附在萃取材料上的有机磷农药进行洗脱,进HPLC检测分析。保证相同的条件,每个过程重复3次,最后结果取平均值。

1.2.5 色谱条件

分析柱为Hypersil ODS C18色谱柱(250 mm×4.6 mm, 5 μm);柱温箱温度为25 ℃。流动相用超纯水作为水相(A),甲醇作为有机相(B);流速为1 mL/min。采用梯度洗脱,具体条件为:0~12 min, 73%B; 12~13 min, 73%B~78%B。紫外检测波长设定为230 nm,样品定量环为20 μL。

## 2 结果与讨论

### 2.1 材料表征

通过扫描电镜观察氧化石墨烯气凝胶的表面形貌(见图1),可以看出,所制备的氧化石墨烯气凝胶表面呈现出层状褶皱的结构,可见石墨烯的片层结构,这种结构使材料本身有较大的表面积,有利于萃取效率的提高。实验进而表征了所制备的氧化石墨烯气凝胶的BET表面积,氧化石墨烯气凝胶的表面积、孔体积和平均宽度分别为740.51 m^2^/g、0.77 cm^3^/g和4.17 nm。

**图 1:**
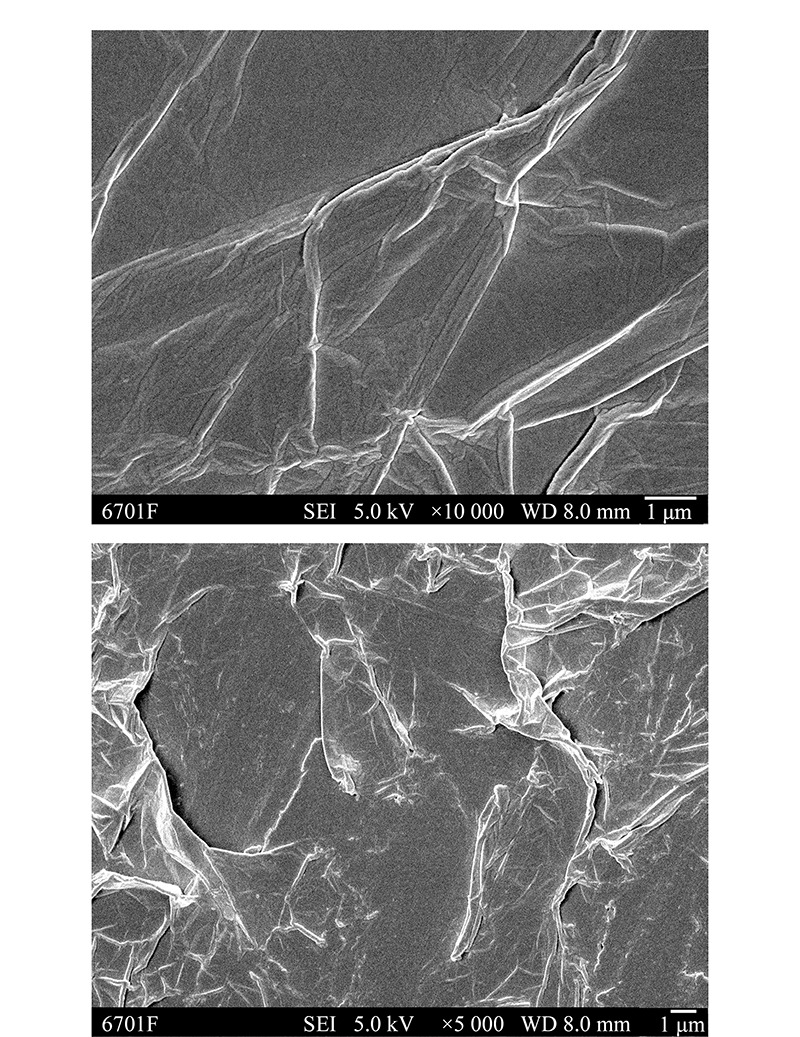
氧化石墨烯气凝胶在不同放大倍数下的扫描电镜图

除此之外,对所制备的氧化石墨烯气凝胶进行了傅里叶红外光谱测定,结果如图2所示。从图中可见,3183 cm^-1^处的峰是氧化石墨烯表面含氧官能团中O-H的伸缩振动峰,2823 cm^-1^和1418 cm^-1^处的峰分别对应的是氧化石墨烯边缘=C-H的伸缩振动峰和弯曲振动峰,1720 cm^-1^处的峰对应的是羧基上C=O的伸缩振动峰,1622 cm^-1^处的峰是氧化石墨烯片层平面上C=C的伸缩振动峰,1043 cm^-1^处的峰是C-O的伸缩振动峰。从中可见氧化石墨烯气凝胶表面的多个含氧官能团未因冷冻被破坏,其极性特征有利于萃取有机磷农药。

**图 2:**
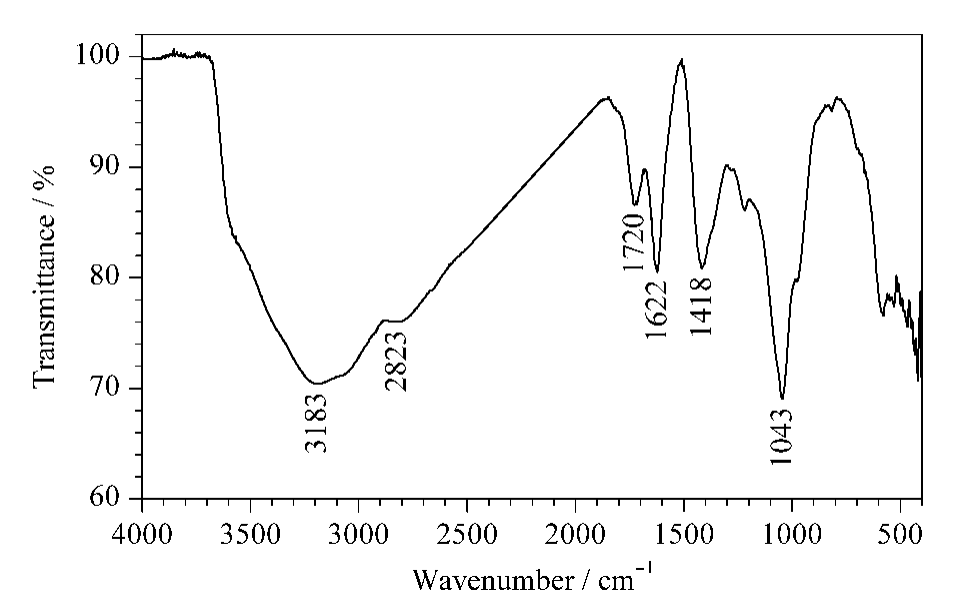
氧化石墨烯气凝胶的红外光谱图

### 2.2 实验条件优化

以4种有机磷农药(辛硫磷、双硫磷、倍硫磷、杀螟硫磷)为考察对象,为获得较优的萃取效果,依次对萃取和洗脱条件进行优化,包括上样体积、上样速率、溶液pH值及洗脱剂类型和体积。实验是在保证其他条件不变的前提下,对其中一个条件进行优化。

2.2.1 上样体积

萃取过程中,在萃取材料保持质量不变的条件下,萃取材料的吸附位点有限,过多的分析物会引起萃取回收率的降低。在本实验中,有机磷农药工作溶液的质量浓度为200 μg/L,依次考察了上样体积为5、10、15、20、40、60 mL时有机磷农药的萃取回收率。从图3a中可以看出,当上样体积低于15 mL时,萃取回收率保持较高值,之后萃取回收率明显降低,所以将此实验的上样体积选择为15 mL。

**图 3:**
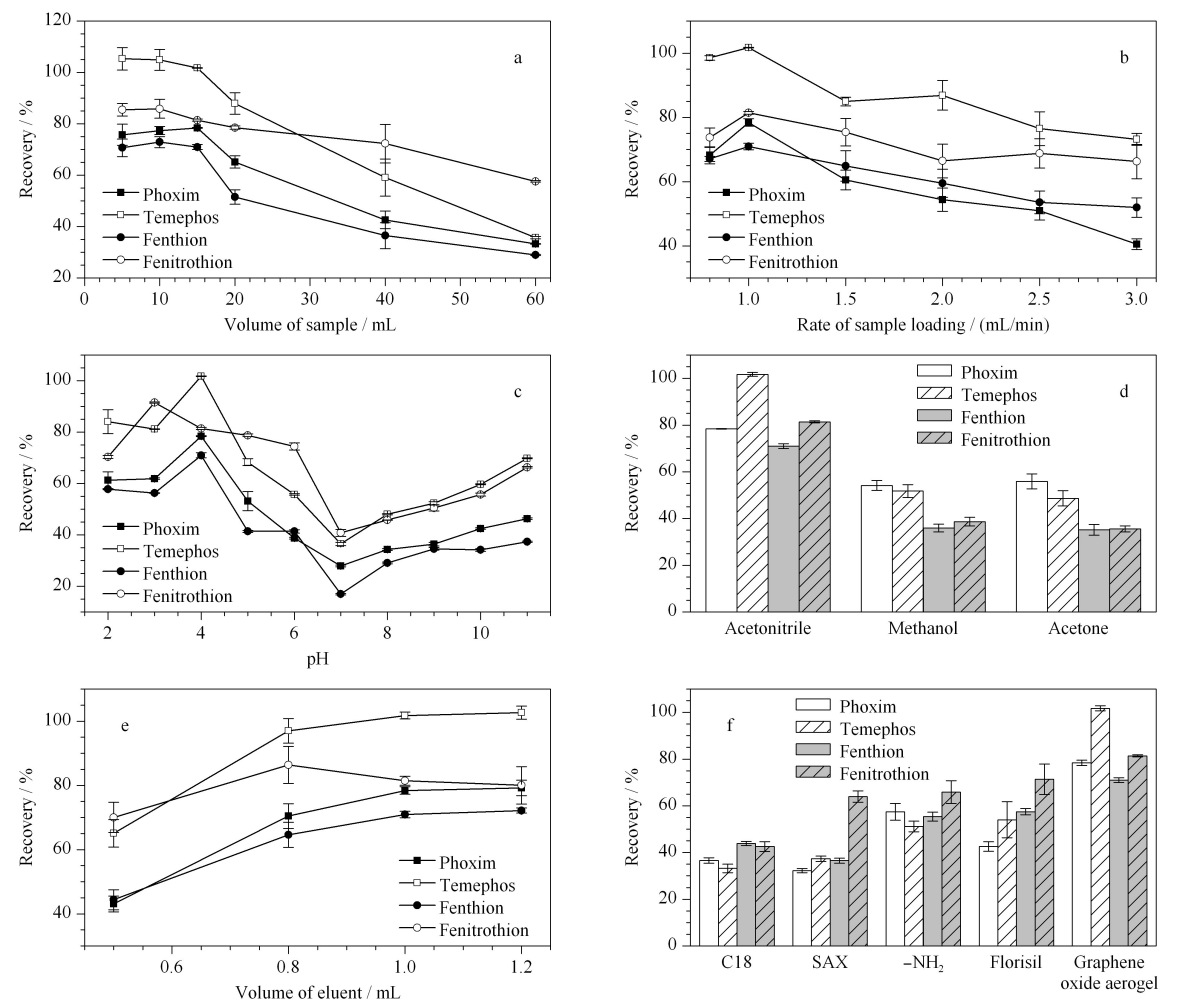
实验条件对萃取回收率的影响(*n*=3)

2.2.2 上样速率

上样速率对萃取回收率的影响也是双向的,上样太快会使得分析物与萃取材料之间接触不充分,太慢会出现分析物被重新洗脱的现象,且造成操作时间过长。此实验考察了0.8~3.0 mL/min的上样速率,如图3b显示,在1.0 mL/min的条件下,萃取回收率达到最大值。

2.2.3 溶液pH值

工作溶液的pH值不仅会影响样品分子的存在状态,也会影响萃取材料表面的电荷,所以本实验对工作溶液的pH值也进行了考察。从图3c中可以看出,样品溶液处于酸性和碱性条件下,萃取回收率较高,这是因为在酸碱性条件下,有机磷农药分子与氧化石墨烯气凝胶之间更容易形成氢键;而且在酸性条件下更有利于有机磷农药分子与氧化石墨烯气凝胶之间静电作用的形成,但pH值太低会造成有机磷农药不易从样品溶液中分离出来,所以萃取回收率随着酸度的降低会升高,从结果中可见,样品溶液pH为4时萃取回收率最大。

2.2.4 洗脱剂类型和体积

洗脱剂的选择是实验参数中重要的环节,它由分析物溶解性及其与萃取材料之间的作用力决定。此实验分别选择了乙腈、甲醇、丙酮对其进行洗脱,结果显示乙腈的洗脱效果最好(见图3d)。乙腈作为有机溶剂,为防止对环境的污染,应尽可能少用,节约溶剂的同时,对分析物富集倍数也会提高,但乙腈必须足够才能保证将有机磷农药充分洗脱下来。此实验进一步考察了乙腈的体积对萃取回收率的影响,从图3e中可以看出,当乙腈体积高于1.0 mL时,回收率较高且变化不大,但当低于1.0 mL时,回收率较低,所以选择1.0 mL乙腈进行洗脱。

### 2.3 萃取性能研究

实验将氧化石墨烯气凝胶填充的固相萃取小柱与商用C18硅胶、氨基柱(-NH_2_)、阳离子交换柱(SAX)、硅酸镁柱(Florisil)(50 mg)等的萃取能力进行比较,实验结果如图3f所示。从图中看到,氧化石墨烯气凝胶对有机磷农药分子的萃取效率要明显高于商用材料,这归功于氧化石墨烯气凝胶的高比表面积和优良的吸附能力,除此之外,氧化石墨烯与有机磷农药分子之间的氢键、*π-π*和静电作用也提高了其萃取能力。基于氧化石墨烯气凝胶的结构特点和吸附特性,此萃取柱具有一定通用性,也可用于一些其他极性或弱极性污染物的萃取。

本实验进一步考察了制备的萃取柱的寿命。将其连续对有机磷农药萃取洗脱25次,使用次数与萃取回收率的关系如图4所示,萃取柱使用15次后,有机磷农药分子的萃取回收率有所降低。因此,该萃取柱使用15次后将其丢弃。

**图 4:**
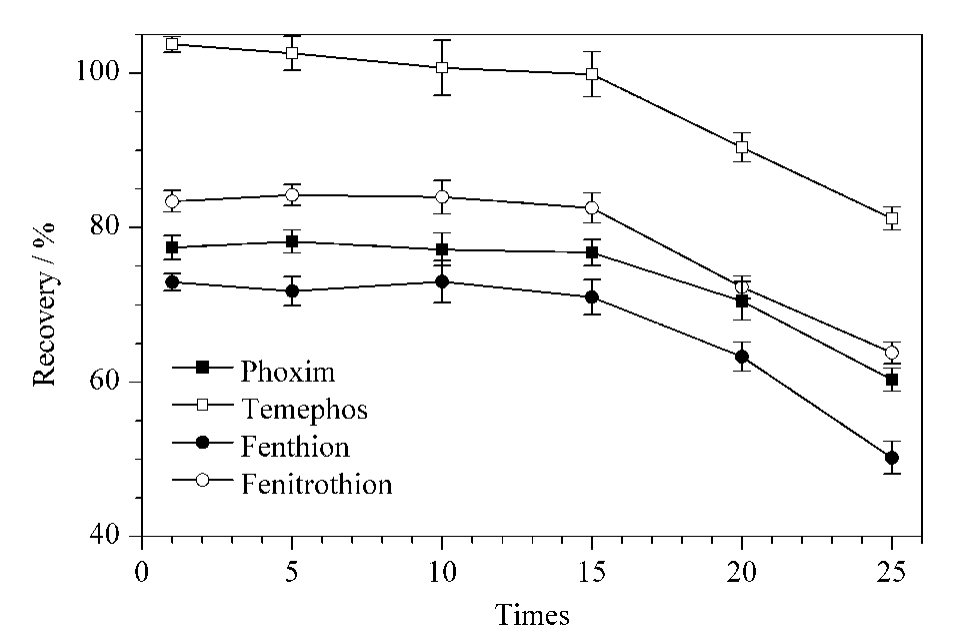
氧化石墨烯气凝胶萃取柱的使用寿命(*n*=3)

### 2.4 方法学考察

配制一系列不同浓度的有机磷农药混合工作溶液,在上述优化的实验条件下,采用氧化石墨烯气凝胶固相萃取柱对上述工作溶液中的有机磷农药进行萃取,并结合HPLC-DAD对分析物进行分离检测。结果列入表1中,从中可见本实验建立的方法对有机磷农药具有良好的线性范围:1~200 μg/L(辛硫磷、双硫磷、倍硫磷)和2~200 μg/L(杀螟硫磷),且线性相关系数(*r*^2^)均大于0.99,检出限≤0.5 μg/L。单根萃取小柱连续萃取5次,有机磷农药的萃取回收率相对标准偏差≤6.5%,然后使用5根萃取小柱萃取分析物,萃取回收率相对标准偏差≤11.3%。

**表 1:** 有机磷农药的线性方程、线性范围、相关系数、检出限、定量限和相对标准偏差

Compound	Linear equation	Linear range/ (μg/L)	r^2^	LOD/ (μg/L)	LOQ/ (μg/L)	RSDs (n=5)/%		
						Single column	Column-to-column	
Phoxim	y=0.2414x+0.4554	1-200	0.9950	0.2	1.0	4.2		11.3
Temephos	y=0.2436x+0.4819	1-200	0.9949	0.5	1.0	4.4		10.2
Fenthion	y=0.2393x-0.0220	1-200	0.9986	0.5	1.0	6.5		7.3
Fenitrothion	y=0.2798x+0.2369	2-200	0.9993	0.5	2.0	2.4		6.5

*y*: peak area; *x*: mass concentration, μg/L.

### 2.5 实际样品应用

将本实验建立的方法应用于苹果皮中有机磷农药的检测分析,选择3个苹果进行分析,每个水平做3个平行样品。从图5中可见,在所选的苹果皮中未检测到4种有机磷农药的存在,随后为考察基质效应对所制备的萃取材料性能的影响,在样品中分别加入20、50和100 μg/L的有机磷农药标准溶液,从表2中可见,所得加标回收率为70.5%~93.6%,相对标准偏差低于10.4%,可以看出在氧化石墨烯气凝胶固相萃取柱-高效液相色谱法检测有机磷农药的过程中,实际样品中的基质对其干扰较小。

**图 5:**
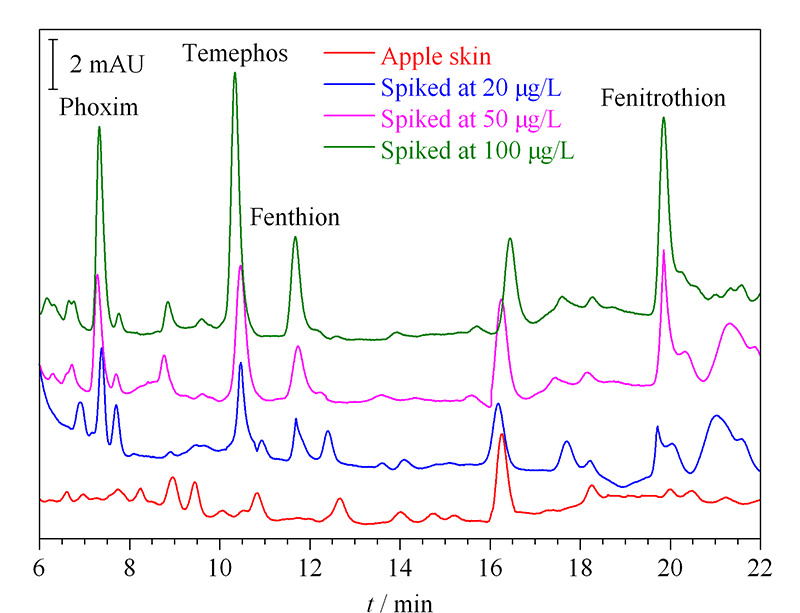
实际样品及加标样品的色谱图

**表 2:** 苹果皮中有机磷农药的含量、加标回收率和相对标准偏差(*n*=3)

Compound	Added/ (μg/L)	Detected/ (μg/L)	Recovery/ %	RSD/ %
Phoxim	20	16.4	82.0	9.6
	50	45.8	91.6	10.4
	100	90.1	90.1	8.5
Temephos	20	14.7	72.5	8.7
	50	41.2	82.4	8.9
	100	89.2	89.2	5.5
Fenthion	20	17.8	89.0	9.2
	50	40.4	80.8	9.4
	100	87.7	87.7	6.3
Fenitrothion	20	14.1	70.5	6.0
	50	46.8	93.6	8.6
	100	91.9	91.9	7.1

## 3 结论

本工作通过冷冻干燥成功制备了氧化石墨烯气凝胶吸附剂,建立了固相萃取-高效液相色谱测定有机磷农药的方法。本方法操作简单,线性范围宽,检出限低,在实际样品中的应用表明氧化石墨烯气凝胶适用于富集食物样品中痕量有机磷农药,对一些弱极性或极性的其他污染物也存在萃取潜力。
